# Collectanea Medica

**Published:** 1812-09

**Authors:** 


### COLLECTANEA MEDICA,

CONSISTING OF

ANECDOTES, FACTS, EXTRACTS, ILLUSTRATIONS, QUERIES, SUGGESTIONS, &c.

RELATING TO THE

*History or the Art of Medicine, and the Auxiliary Sciences.*

### *Teaching the Deaf and Dumb to speak.*

THIS very important art was first contrived by Dr. Wallis, whose versatile talents and industry displayed themselves in so many ways, and gave birth to so many admirable inventions. He has himself given us an account of the method which he practised. The person on whom he began the trial was Mr. Daniel Whalley, who had once been able to speak, but, having lost his hearing when only five years of age, he had gradually, in consequence, left off speaking, and forgotten all language. The task of teaching a deaf person to speak consists of two parts, quite independent of each other, and rendered on that account more difficult. The first consists in teaching the scholar the position of the tongue, tongue, and other organs of speech, in pronouncing the different sounds; the second, in making him acquainted with the meaning of words, and with the use of language. The commencement of both of these parts is most difficult. The position of the organs is explained as precisely as possible by signs, and, when a particular sound is once attained, the scholar, by frequent repetition, is prevented from forgetting it. In explaining the meaning of words, Dr. Wallis arranged the most material words in classes, and pointed out their meaning, either by showing the objects of which they were the names, or by some other equivalent means. In about two months, Mr. Whalley was able to pronounce most words, and to understand an English book on common subjects. He was on the 21st of May, 1662, present at a meeting of the Royal Society, and in their presence, and to their great satisfaction, pronounced distinctly enough such words as by the company were proposed to him; and, though not altogether with the usual tone and accent, yet so as to be easily understood. He did the same thing before King Charles II., Prince Rupert, and several of the nobility. In the space of one year, which was the whole time of his stay with Dr. Wallis, he had read over a great part of the English Bible, and had attained so much skill as to be able to express himself intelligibly in ordinary affairs, to understand letters written to him, and to write answers to them, though not elegantly, yet so as to be understood; and in presence of foreigners he pronounced the most difficult words of their language that could be proposed to him.*—*History of the Royal Society, by Dr. Thompson.*

The merit and character of Dr. Wallis are fully admitted; but it is a question if *he* was the "*contriver*" of the art of teaching the deaf and dumb to speak. Mr. Whalley was under the tuition of Dr. Wallis one year, (1661 probably,) and in 1662 he was exhibited to the Royal Society. But an ingenious English physician, a man of deep and laborious research, had published before 1653 a treatise which he called "PHILOCOPHUS, or the Deafe and Dumbe Man's Friend;" and had composed before the above date, "*The Academy for the Deafe and Dumbe: being the manner of the operation to bring those who are so borne, to heare the sound of Words with the Eyes, and thence to learne to speake with the Tongue.*" Beside the above, this writer had also a "*Tractatus de removendis Loquilæ impedimentis;*" and a "*Tractatus de removendis Auditoris impedimentis.*" With the PHILOCOPHUS, published many years before Dr. Wallis in-

* Phil. Trans. 1670. Vol. v. p. 1087; and 1698. Vol. xx. p. 353.

structed structed Mr. Whalley, or laid before the public any account of his method; and, with the other treatises ready for the press, and advertised in 1653, it cannot be allowed that Dr. Wallis was the original contriver of the important art of teaching the deaf and dumb to speak, an account of which he first published in 1670.

---

### *Healthiness of London.*

One of the very first things that strike one is the surprising difference in the healthiness of London at present, notwithstanding its great increase of population, and what it was during the 17th century. At present the number of inhabitants in London, and the contiguous villages, which in fact make a part of it, is 1,090,000. We do not know exactly what it was in the 17th century, but, as it has been increasing ever since, and, as in the year 1753, the number of inhabitants did not exceed 750,000,* we shall not probably err very much if we reckon the inhabitants of London at the revolution, in 1688, about half a million. Yet, in the years 1685, 1686, and 1687, the births and deaths were to each other as follows:†

| | Births. | Deaths. |
|---|---|---|
| 1685 | 14,730 | 23,222 |
| 1686 | 14,694 | 22,609 |
| 1687 | 14,951 | 21,460 |

So that, at this period, the deaths exceeded the births by no less a quantity than 7,639, or more than one half of the whole births. At present the number of births exceed that of the deaths. This must be owing to the different mode of living, and the improvement in the width of the streets, and in cleanliness. The same improvements having taken place in every part of Great Britain, it is probable that the value of human life is rather increased in this island.—*Ibid.*

---

### *On a gaseous Compound of carbonic Oxide and Chlorine. By* JOHN DAVY, Esq. *Communicated by* Sir HUMPHRY DAVY, Knt. LL.D. Sec. R.S.

Since the influence of electricity and solar light, as chemical agents, are analogous in many respects, and as the former produces no change in a mixture of carbonic oxide and chlorine, it was natural to infer the same respecting the latter. MM. Gay Lussac and Thenard assert that this is the case; they say that they have exposed a mixture of carbonic

* Phil. Trans. 1754. Vol. xlviii. p. 788.

† Phil. Trans. 1685. Vol. xv. p. 1245; and Vol. xvii. p. 445.

 oxide oxide and chlorine, under all circumstances, to light, without observing any alteration to take place.* Mr. Murray has made a similar statement.†

Having been led to repeat this experiment, from some objections made by the last-mentioned gentleman to the theory of my brother, Sir Humphry Davy, concerning chlorine, I was surprised at witnessing a different result.

The mixture exposed, consisted of about equal volumes of chlorine and carbonic oxide; the gases had been previously dried over mercury by the action of fused muriate of lime, and the exhausted glass globe into which they were introduced from a receiver with suitable stopcocks, was carefully dried. After exposure for about a quarter of an hour to bright sunshine, the color of the chlorine had entirely disappeared; the stopcock belonging to the globe being turned in mercury recently boiled, a considerable absorption took place, just equal to one half the volume of the mixture, and the residual gas possessed properties perfectly distinct from those belonging either to carbonic oxide or chlorine.

Thrown into the atmosphere, it did not fume. Its odor was different from that of chlorine, something like that which one might imagine would result from the smell of chlorine combined with that of ammonia, yet more intolerable and suffocating than chlorine itself, and affecting the eyes in a peculiar manner, producing a rapid flow of tears, and occasioning painful sensations.

Its chemical properties were not less decidedly marked than its physical ones.

Thrown into a tube full of mercury containing a slip of dry litmus paper, it immediately rendered the paper red.

Mixed with ammoniacal gas, a rapid condensation took place, a white salt was formed, and much heat was produced.

The compound of this gas and ammonia was a perfect neutral salt, neither changing the color of turmeric or litmus; it had no perceptible odor, but a pungent saline taste; it was deliquescent, and of course very soluble in water; it was decomposed by the sulphuric, nitric, and phosphoric, acids, and also by liquid muriatic acid; but it sublimed unaltered in the muriatic, carbonic, and sulphureous, acid gases, and dissolved without effervescing in acetic acid. The products of its decomposition collected over mercury were found to be the carbonic and muriatic acid gases; and in the experiment with concentrated sulphuric acid when accurate

* Recherches Physico-Chimiques, tom. ii. p. 150.

† Nicholson's Journal, vol. xxx. p. 227.

results could be obtained, these two gases were in such proportions, that the volume of the latter was double that of the former.

I have ascertained by repeated trials, both synthetical and analytical, that the gas condenses four times its volume of the volatile alkali, and I have not been able to combine it with a smaller proportion.

Tin fused in the gas in a bent glass tube over mercury, by means of a spirit lamp, rapidly decomposed it; the liquor of Libavius was formed; and, when the vessel had cooled, there was not the least change of the volume of the gas perceptible; but the gas had entirely lost its offensive odor, and was merely carbonic oxide; for like carbonic oxide it burnt with a blue flame, afforded carbonic acid by its combustion, and was not absorbable by water.

The effects of zinc, antimony, and arsenic, heated in the gas, were similar to those of tin; compounds of these metals and chlorine were formed, and carbonic oxide in each experiment was liberated equal in volume to the gas decomposed. In each instance the action of the metal was quick; the decomposition being completed in less than ten minutes; but, though the action was rapid, it was likewise tranquil; no explosion ever took place, and none of the metals became ignited or inflamed.

The action even of potassium heated in the gas was not violent. But, from the great absorption of gas, and from the precipitation of carbon indicated by the blackness produced, not only the new gas, but likewise the carbonic oxide, appeared to be decomposed.

The white oxide of zinc heated in the gas quickly decomposed it, just as readily indeed as the metal itself; there was the same formation of the butter of zinc; but, instead of carbonic oxide being produced, carbonic acid was formed; and, as usual, there was no change of volume.

The protoxide of antimony fused in the gas rapidly decomposed it; the butter of antimony and the infusible peroxide were formed; there was no change of the volume of the gas, and the residual gas was carbonic oxide.

Sulphur and phosphorus sublimed in the gas, produced no apparent change; the volume of the gas was unaltered, and its characteristic smell was undiminished.

Mixed with hydrogen or oxygen singly, the gas was not inflamed by the electric spark, but mixed with both, in proper proportions, viz. two parts in volume of the former and one of the latter to two parts of the gas, a violent explosion was produced, and the muriatic and carbonic acid gases were formed.

The

The gas transferred to water was quickly decomposed, the carbonic and muriatic acids were formed, as in the last experiment, and the effect was the same even when light was excluded.

From the mode of the formation of the gas, and the condensation that takes place at the time, from the results of the decomposition of its ammoniacal salt, and from the analysis of the gas by metals and metallic oxides, it appears to be a compound of carbonic oxide and chlorine condensed into half the space which they occupied separately.

And, from its combining with ammonia, and forming with this alkali a neutral salt, and from its reddening litmus, it seems to be an acid. It is similar to acids in other respects; in decomposing the dry sub-carbonate of ammonia, one part in volume of it expelling two parts of carbonic acid gas; and in being itself not expelled from ammonia by any of the acid gases, or by acetic acid. Independent of these circumstances, were power of saturation to be taken as the measure of affinity, the attraction of this gas for ammonia must be allowed to be greater than that of any other substance, for its saturating power is greater; no acid condenses so large a proportion of ammonia; carbonic acid only condenses half as much, and yet does not form a neutral salt. The great saturating and neutralizing powers of this gas are singular circumstances, and particularly singular when compared with those of muriatic acid gas.

In consequence of its being decomposed water, I have not been able to ascertain whether it is capable of combining with the fixed alkalies. Added to solutions of these substances it was absorbed, and carbonic acid gas was disengaged by an acid.

I have made the experiment on the native carbonates of lime and barytes, but the gas did not decompose these bodies. This indeed might be expected, since quick-lime, I find, does not absorb the gas: a cubic inch of it, exposed to the action of lime in a tube over mercury, was only diminished in two days to nine-tenths of a cubic inch, and no further absorption was afterwards observed to take place. But even this circumstance does not demonstrate that the gas has no affinity for lime, and is not capable of combining with it; for, on making a similar experiment with carbonic acid, substituting this gas for the new compound, the result was the same; in two days only about one-tenth of a cubic inch was absorbed.

Though the gas is decomposed by water, yet it appears to be absorbed unaltered by common spirits of wine, which contains so considerable a quantity of water; it imparted its peculiar peculiar odor to the spirit, and its property of affecting the eyes; five measures of the spirit condensed sixty measures of the gas.

It is also absorbed by the fuming liquor of arsenic, and by the oxymuriate of sulphur.

The former appeared to require for saturation ten times its own volume; six measures of the liquor condensed about sixty of the gas. The liquor thus impregnated was thrown into water, and a pretty appearance was produced by the sudden escape of bubbles of the gas: had not its intolerable smell convinced me that the gas was unaltered, I should not have conceived that it could pass through water undecomposed.

I cannot account for the assertion of MM. Gay Lussac and Thenard and of Mr. Murray, that oxymuriatic gas does not, when under the influence of light, exert any action on carbonic oxide: I was inclined at first to suppose that the difference between their results and mine, might be owing to their not having exposed the gases together to bright sunshine; but I have been obliged to relinquish this idea, since I have found that bright sunshine is not essential, and that the combination is produced in less than twelve hours by the indirect solar rays, light alone being necessary.

The formation of the new gas may be very readily witnessed, by making a mixture of dry carbonic oxide and chlorine in a glass tube over mercury: if light be excluded, the chlorine will be absorbed by the mercury, the carbonic oxide alone remaining; but, if bright sunshine be immediately admitted when the mixture first is made, a rapid ascension of the mercury will take place, and in less than a minute the color of the chlorine will be destroyed, and in about ten minutes the condensation will have ceased, and the combination of the two gases will be complete.

It is requisite that the gases should be dried for forming this compound; if this precaution is neglected, the new gas will be far from pure; it will contain a considerable admixture of the carbonic and muriatic acid gases, which are produced in consequence of the decomposition of hygrometrical water. Indeed there is considerable difficulty in procuring the new gas tolerably pure; a good air-pump is required, and perfectly tight stopcocks, and dry gases, and dry vessels.

I have endeavored to procure the gas, by passing a mixture of carbonic oxide and chlorine through an earthenware tube heated to redness, but without success.

The specific gravity of the gas may be inferred from the specific gravities of its constituent parts jointly with the condensation that takes place at their union. According to Cruickshank,

Cruickshank, 100 cubic inches of carbonic oxide weigh 29·6 grains; and, according to Sir Humphry Davy, 100 of chlorine are equal to 76·37 grains: hence, as equal volumes of these gases combine, and become so condensed as to occupy only half the space they before filled, it follows that 100 cubic inches of the new compound gas are equal to 105·97 grains. Thus this gas exceeds most others as much in its density as it does in its saturating power.

To ascertain whether chlorine has a stronger affinity for hydrogen than for carbonic oxide, I exposed a mixture of the three gases in equal volumes to light. Both the new compound and muriatic acid gas were formed, and the affinities were so nicely balanced, that the chlorine was nearly equally divided between them. And that the attraction of chlorine for both these gases is nearly the same, appears to be confirmed by muriatic acid not being decomposed by carbonic oxide, or the new gas by hydrogen.

The chlorine and carbonic oxide are, it is evident from these last facts, united by strong attractions; and, as the properties of the substance as a peculiar compound are well characterised, it will be necessary to designate it by some simple name. I venture to propose that of phosgene, or phosgene gas; from Φως, light, and γινομαι, to produce, which signifies formed by light; and as yet no other mode of producing it has been discovered.

I have exposed mixtures consisting of different proportions of chlorine and carbonic acid to light, but have obtained no new compound.

The proportions in which bodies combine appear to be determined by fixed laws, which are exemplified in a variety of instances, and particularly in the present compound. Oxygen combines with twice its volume of hydrogen and twice its volume of carbonic oxide to form water and carbonic acid, and with half its volume of chlorine to form euchlorine; and chlorine reciprocally requires its own volume of hydrogen and its own volume of carbonic oxide to form muriatic acid and the new gas.

This relation of proportions is one of the most beautiful parts of chemical philosophy, and that which promises fairest, when prosecuted, to raise chemistry to the state and certainty of a mathematical science.—*Phil. Trans. p.* 1. 1812.

---

*A Narrative of the Eruption of a Volcano in the Sea off the Island of St. Michael. By* JAMES TILLARD, Esq. Captain in the Royal Navy.

Approaching the island of St. Michael's, on Sunday the 12th of June, 1811, in his majesty's sloop Sabrina, under my command, command, we occasionally observed, rising in the horizon, two or three columns of smoke, such as would have been occasioned by an action between two ships, to which cause we universally attributed its origin. This opinion was, however, in a very short time changed, from the smoke increasing and ascending in much larger bodies than could possibly have been produced by such an event; and, having heard an account, prior to our sailing from Lisbon, that in the preceding January or February a volcano had burst out within the sea near St. Michael's, we immediately concluded that the smoke we saw proceeded from that cause, and on our anchoring the next morning in the road of Ponta del Gada, we found this conjecture correct as to the cause, but not to the time; the eruption of January having totally subsided, and the present one having only burst forth two days prior to our approach, and about three miles distant from the one before alluded to.

Desirous of examining as minutely as possible a contention so extraordinary between two such powerful elements, I set off from the city of Ponta del Gada on the morning of the 14th, in company with Mr. Read, the consul-general of the Azores, and two other gentlemen. After riding about twenty miles across the north-west end of the island of St. Michael's, we came to the edge of a cliff, from whence the volcano burst suddenly upon our view in the most terrific and awful grandeur. It was only a short mile from the base of the cliff, which was nearly perpendicular, and formed the margin of the sea; this cliff being, as nearly as I could judge, from three to four hundred feet high. To give you an adequate idea of the scene by description is far beyond my powers, but for your satisfaction I shall attempt it.

Imagine an immense body of smoke rising from the sea, the surface of which was marked by the silvery ripling of the waves, occasioned by the light and steady breezes incidental to those climates in summer. In a quiescent state, it had the appearance of a circular cloud revolving on the water like an horizontal wheel, in various and irregular involutions, expanding itself gradually on the lee side, when suddenly a column of the blackest cinders, ashes, and stones, would shoot up in form of a spire at an angle of from ten to twenty degrees from a perpendicular line, the angle of inclination being universally to windward: this was rapidly succeeded by a second, third, and fourth, each acquiring greater velocity, and overtopping the other till they had attained an altitude as much above the level of our eye, as the sea was below it.

As the impetus with which the columns were severally propelled diminished, and their ascending motion had nearly ceased, they broke into various branches resembling a groupe of pines, these again forming themselves into festoons of white feathery smoke, in the most fanciful manner imaginable, intermixed with the finest particles of falling ashes, which at one time assumed the appearance of innumerable plumes of black and white ostrich feathers surmounting each other; at another, that of the light wavy branches of a weeping willow.

During these bursts, the most vivid flashes of lightning continually issued from the densest part of the volcano; and the cloud of smoke now ascending to an altitude much above the highest point to which the ashes were projected, rolled off in large masses of fleecy clouds, gradually expanding themselves before the wind in a direction nearly horizontal, and drawing up to them a quantity of water-spouts, which formed a most beautiful and striking addition to the general appearance of the scene.

That part of the sea where the volcano was situated, was upwards of thirty fathoms deep, and at the time of our viewing it the volcano was only four days old. Soon after our arrival on the cliff, a peasant observed he could discern a peak above the water; we looked, but could not see it; however, in less than half an hour it was plainly visible, and before we quitted the place, which was about three hours from the time of our arrival, a complete crater was formed above the water, not less than twenty feet high on the side where the greatest quantity of ashes fell; the diameter of the crater being apparently about four or five hundred feet.

The great eruptions were generally attended with a noise like the continued firing of cannon and musquetry intermixed, as also with slight shocks of earthquakes, several of which having been felt by my companions, but none by myself, I had become half sceptical, and thought their opinion arose merely from the force of imagination: but, while we were sitting within five or six yards of the edge of the cliff, partaking of a slight repast which had been brought with us, and were all busily engaged, one of the most magnificent bursts took place which we had yet witnessed, accompanied by a very severe shock of an earthquake. The instantaneous and involuntary movement of each was to spring upon his feet, and I said, "This admits of no doubt." The words had scarce passed my lips, before we observed a large portion of the face of the cliff, about fifty yards on our left, falling, which it did with a violent crash. So soon as our first consternation had a little subsided, we removed about ten or a dozen yards further from the edge of the cliff, and finished our dinner.

On

On the succeeding day, June 15th, having the consul and some other friends on-board, I weighed, and proceeded with the ship towards the volcano, with the intention of witnessing a night view; but in this expectation we were greatly disappointed, from the wind freshening and the weather becoming thick and hazy, and also from the volcano itself being clearly more quiescent than it was the preceding day. It seldom emitted any lightning, but occasionally as much flame as may be seen to issue from the top of a glass-house or foundery chimney.

On passing directly under the great cloud of smoke, about three or four miles distant from the volcano, the decks of the ship were covered with fine black ashes, which fell intermixt with small rain. We returned the next morning, and late on the evening of the same day I took my leave of St. Michael's to complete my cruize.

On opening the volcano clear of the north-west part of the island, after dark on the 16th, we witnessed one or two eruptions that, had the ship been near enough, would have been awfully grand. It appeared one continued blaze of lightning; but the distance which it was at from the ship, upwards of twenty miles, prevented our seeing it with effect.

Returning again towards St. Michael's on the 4th of July, I was obliged, by the state of the wind, to pass with the ship very close to the island, which was now completely formed by the volcano, being nearly the height of Matlock High Tor, about eighty yards above the sea. At this time it was perfectly tranquil; which circumstance determined me to land, and explore it more narrowly.

I left the ship in one of the boats, accompanied by some of the officers. As we approached, we perceived that it was still smoking in many parts, and upon our reaching the island found the surf on the beach very high. Rowing round to the lee side, with some little difficulty, by the aid of an oar, as a pole, I jumped on shore, and was followed by the other officers. We found a narrow beach of black ashes, from which the side of the island rose in general too steep to admit of our ascending; and, where we could have clambered up, the mass of matter was much too hot to allow our proceeding more than a few yards in our ascent.

The declivity below the surface of the sea was equally steep, having seven fathoms water scarce the boat's length from the shore, and at the distance of twenty or thirty yards we sounded twenty-five fathoms.

From walking round it in about twelve minutes, I should judge that it was something less than a mile in circumference; but the most extraordinary part was the crater, the mouth of which, on the side facing St. Michael's, was nearly level with the sea. It was filled with water, at that time boiling, and was emptying itself into the sea by a small stream about six yards over, and by which I should suppose it was continually filled again at high water. This stream, close to the edge of the sea, was so hot, as only to admit the finger to be dipped suddenly in, and taken out again immediately.

It appeared evident, by the formation of this part of the island, that the sea had, during the eruptions, broke into the crater in two places, as the east side of the small stream was bounded by a precipice, a cliff between twenty and thirty feet high, forming a peninsula of about the same dimensions in width, and from fifty to sixty feet long, connected with the other part of the island by a narrow ridge of cinders and lava, as an isthmus of from forty to fifty feet in length, from which the crater rose in the form of an amphitheatre.

This cliff, at two or three miles distance from the island, had the appearance of a work of art resembling a small fort or block house. The top of this we were determined, if possible, to attain; but the difficulty we had to encounter in doing so was considerable; the only way to attempt it was up the side of the isthmus, which was so steep, that the only mode by which we could effect it, was by fixing the end of an oar at the base, with the assistance of which we forced ourselves up in nearly a backward direction.

Having reached the summit of the isthmus, we found another difficulty, for it was impossible to walk upon it, as the descent on the other side was immediate, and as steep as the one we had ascended; but by throwing our legs across it, as would be done on the ridge of a house, and moving ourselves forward by our hands, we at length reached that part of it where it gradually widened itself and formed the summit of the cliff, which we found to have a perfectly flat surface, of the dimensions before stated.

Judging this to be the most conspicuous situation, we here planted the Union, and left a bottle sealed up containing a small account of the origin of the island, and of our having landed upon it, and naming it Sabrina Island.

Within the crater I found the complete skeleton of a guard fish, the bones of which, being perfectly burnt, fell to pieces upon attempting to take them up; and, by the account of the inhabitants on the coast of St. Michael's, great numbers of fish had been destroyed during the early part part of the eruption, as large quantities, probably suffocated or poisoned, were occasionally found drifted into the small inlets or bays.

The island, like other volcanic productions, is composed principally of porous substances, and generally burnt to complete cinders, with occasional masses of a stone, which I should suppose to be a mixture of iron and lime-stone, but have sent you specimens to enable you to form a better judgment than you possibly can by any description of mine.—*Phil. Trans. p.* 1. 1812.

---

### *Analysis of Deadly Nightshade, Atropa Belladonna; by* MR. VAUQUELIN.

The experiments I am about to relate were instituted for the purpose of knowing whether this plant, which is of the same family as tobacco, contained the acrid principle that we found in the latter; but we shall see below that it does not exist in it. However, I availed myself of this opportunity to examine the properties of the matter in this plant, which, according to the physicians, is narcotic.

1. The expressed and filtered juice of belladonna has a pretty deep brown color, and a bitter nauseous taste.

It is copiously coagulated by heat, and by an aqueous infusion of gall.

2. The substance coagulated by heat in the juice of belladonna is of a yellowish grey, becomes black by dessication, and presents a smooth polished fracture like that of resins. It burns with decrepitation, softening, and smoke, which has the same smell as horn similarly treated.

3. The juice of belladonna, distilled till it is reduced to the consistence of a liquid extract, yielded a water which had only a flat herbaceous taste, and nothing of the acrimony of that of tobacco. The only re-agent, of all we tried, that rendered it slightly turbid, was acetate of lead.

4. The juice reduced to the consistence of an extract being treated with alcohol, part dissolved in it, and the solution deposited on cooling crystals of nitrate of potash, and a little muriate of the same base.

The alcohol, separated from these crystals and evaporated, left as a residuum a brownish yellow matter, of a very bitter and nauseous taste, which, taken up a second time by highly dephlegmated alcohol, left a fresh quantity of insoluble matter, and still deposited a few crystals of the same salt.

The matter being divested as much as possible by this process of the greater part of the nitre, and of the substance insoluble soluble in alcohol, I evaporated the alcohol, and subjected its residuum to the following experiments:

1. It dissolves abundantly and speedily in water, and is even deliquescent in the air.

2. The solution is of a yellowish brown, and has a very bitter and very disagreeable taste.

3. It reddens litmus paper very deeply.

4. It is copiously precipitated by alcoholic tincture of galls, and not by acetate of lead, if the latter be sufficiently diluted with water; but, as it contains a little muriate of potash, it would precipitate the acetate of lead without this precaution.

5. This solution, when mixed with sulphuric acid, emitted a very evident smell of acetic acid.

6. The same solution, on the addition of nitrate of silver, threw down a true muriate of silver.

7. Caustic potash produces from the solution a fetid smell very similar to that of stale lye, in which linen has been washed, and which is beginning to putrefy. Ammoniacal vapours too arise, which may be rendered sensible by weak nitric acid held at a little distance from the mixture.

8. The addition of a few drops of sulphate of iron renders the solution of a much deeper color.

9. The extract itself, exposed on burning coals, swells up, and emits pungent and acrid fumes, in which the smell cannot distinguish ammonia.

From the effects produced on the solution of extract of belladonna by the various tests employed above, we may conclude that it contains, 1, a free acid; 2, an alkaline muriate; 3, a small quantity of an ammoniacal salt.

The acid that exists in it must be the acetic, since sulphuric acid elicits the smell of this acid, and acetate of lead occasions no precipitate, which it would, if the acid were the malic, tartarous, or oxalic. Part of this acid must be combined with potash; and it is this, no doubt, that communicates to the extractive mass the property of attracting the moisture of the air.

But neither these salts, nor these acids (this acid), impart to the matter its poisonous qualities. These unquestionably reside in the vegetable substance itself. What then is the order of composition that makes thus, with the same principles, both our food and such deadly poisons? This is one of the barriers that chemistry has not yet been able to overstep, and unfortunately beyond this barrier lie secrets of the utmost importance to mankind. Wanting, therefore, the means which at some future period may give us a precise knowledge of the differences that exist between vegetable compounds possessing such opposite properties, we must have recourse to observation of their effects.

One of the methods that appeared to us best adapted to elucidate the nature of that substance in belladonna, which is soluble in alcohol, was its decomposition by fire. Accordingly I introduced 2·7 gr. (41·7 grs.) into a glass retort, and heated it gradually, till the water of solution had been distilled over by a very strong heat. A yellow ammoniacal liquid passed over, and afterward a thick oil, which had a very singular disagreeable smell.

On examining the liquid product, I found a great deal of free ammonia, though there was some in combination, for the addition of a few drops of caustic potash rendered the ammoniacal smell much stronger. The oil was black, very thick, and very acrid.

The coal left in the retort weighed 1 gr. (15·45 grs.) It had an alkaline and prussiate taste. Washed with boiling water it yielded a lixivium, which, being mixed with sulphate of iron, produced a very considerable quantity of prussian blue, considering the small quantity of matter employed. The coal, after having been lixiviated and dried, still weighed 7 dec. (10 81 grs.)

This quantity of coal, exclusive of what was encrusted on the retort by the violence of the fire, which I was unable to separate, is larger than is furnished by most other vegetable matters, that I have yet had an opportunity of distilling: for the 2·7 gr. (41·7 grs.) certainly contained more than 7 dec. (10·81 grs.) of water, beside nitrate and acetate of potash.

It appears too, that it contains a large quantity of nitrogen and hidrogen, since it yielded by distillation a great deal of ammonia, prussic acid, and oil. But, as this matter might contain a little nitrate, I suspected that part at least of the nitrogen forming the ammonia and prussic acid, was produced from the nitric acid.

To clear up this doubt, I mixed 6 gr. (92·67 grs.) of gum arabic, in which there is supposed to be nitrogen, with a tenth of saltpetre; and, after having distilled, examined the products. The liquid that came over was in fact ammoniacal; and its smell became stronger on the addition of potash, which shows that an acid was formed at the same time with the alkali.

The coal remaining in the retort, which weighed 2 gr. (30·89 grs.) and was extremely pyrophoric, contained prussiate of potash, like that of my matter. But though I employed in this experiment three times as much gum, and probably more saltpetre, the mixture did not furnish so large a quantity quantity of ammonia, or of prussic acid, as the nauseous principle of belladonna.

If we admit, therefore, that the saltpetre contained in the 2 gr. of this principle gave rise to some prussic acid and ammonia, we ought not to infer that the vegetable matter in question furnished none. That it did, is the more probable, because its solution was precipitated by infusion of galls. Be it as it may, this experiment shews, that it is difficult to judge, from distillation, whether organic matter containing saltpetre be of a vegetable or animal nature.

The results of this analysis, though hitherto very rude, are sufficient, however, to show that the substance which constitutes the subject of them, contains a great deal of charcoal, hidrogen, and nitrogen, and but little oxigen, if we may judge from the small quantity of carbonic acid formed during its decomposition by fire.

From what has been said may we be allowed to suppose, that the narcotic effects which belladonna produces in the animal economy, are owing to the superabundance of combustible radicals, and particularly to that of the charcoal over that of the oxigen in the principle of this plant soluble in spirit of wine? Without pretending to assert this, it is nevertheless certain, that all the vegetable matters which produce analogous effects, are rich in charcoal, hidrogen, and nitrogen, while substances greatly oxigenated produce contrary effects.

It must be confessed too, that a great many vegetable products, equally abundant in these two principles, do not possess the same qualities; but the nitrogen, which is always found associated with hydrogen and charcoal in the somniferous plants, does not exist, at least in similar quantity, in the others.

*Examination of the part of belladonna insoluble in alcohol.*—1. This matter, dissolved in water, communicates to it the property of frothing when shaken.

2. The solution is copiously precipitated by aqueous infusion of galls.

3. Nitrate of barytes causes in it a precipitate partly soluble in nitric acid.

4. Muriate of lime produces a precipitate wholly soluble in nitric acid.

5. The solution reddens litmus paper.

6. Nitrate of silver produces in it no effect.

7. Burned in a crucible it leaves an alkaline and hepatic coal.

From these effects we may conclude, that this part of belladonna is composed of an animal matter, of sulphate of potash, potash, of acidulous oxalate of potash, probably of nitrate, and that it contains no muriate.

We may conclude too from these effects, that no earthy salts are present in it, since muriate of lime, as well as nitrate of barytes, produces in it a precipitate.

I satisfied myself by trials made on a larger scale, that the precipitates occasioned in the solution of the substance in question by nitrate of barytes, were, the first oxalate of lime, the second sulphate of barytes.

The oxalate of lime had carried down with it a large quantity of animal matter, which gave it a brown color. This indicates, that this salt has a powerful attraction for animal matter; and explains why mulberry calculi, which are known to be composed of oxalate of lime, have a much deeper color than other calculi.

After having precipitated successively, as I have said, the sulphate of potash, and acidulous oxalate of potash, I evaporated the liquor, which was still colored, and contained nitrate of potash and muriate of lime; and I treated it with nitric acid, to know whether it contained any gum: but, as I could not obtain an atom of sacchlactic acid, I concluded that it contained none. It was formed only of oxalic acid, and a yellow matter. This substance appeared then to be entirely of an animal nature.

From what has been said, we find that the juice of belladonna contains the following matters:

1. An animal substance, which is partly coagulated by heat, and partly remains dissolved in the juice by means of the free acetic acid present in it.

2. A substance soluble in spirit of wine, which has a bitter and nauseous taste, by combining with tannin becomes insoluble, and furnishes ammonia when decomposed by fire.

3. Several salts with base of potash, namely, a great deal of nitrate, some muriate, some sulphate, acidulous oxalate, and acetate.

The substance of the belladonna, from which the juice had been expressed, having been washed with hot water, dried, and then burned, left ashes composed of a pretty large quantity of lime, phosphate of lime, iron, and silex.

This lime announces that the plant contained oxalate of lime, which had been decomposed by the fire.

There can be no doubt that it is the matter in belladonna, soluble in alcohol, which alone produces its deleterious effect on the animal economy; for it is the only sapid part, and the well known effects of all the other matters accompanying it in no respect resemble those of the plant.

To place this beyond doubt, I gave a middle-sized dog a certain quantity of this principle mixed with his food.

A quarter after twelve I made this dog take 1 gr. (15·45 grs.) of the extract, rolled up in 10 gr. (154·5 grs.) of bread and meat made into a paste.

In three quarters of an hour the animal appeared inclined to sleep; he held his head down, and seemed unable to support it; he lay down several times with his head on the ground; his paws were slightly convulsed; his jaws moved for some time, as if he were chewing. These effects continued about three quarters of an hour, but nothing farther ensued, and the dog resumed his ordinary manners.

At two o'clock in the afternoon I gave him 2 gr. (30·89 grs.) of the extract in 12 grs. of paste. The effects were renewed; but they were slighter, and of shorter duration.

At three o'clock I made him swallow 4 gr. (61·78 grs.) of the same extract, with about 30 gr. (363 grs.) of paste.

A few minutes after he was seized with a continual but uncertain and difficult movement, chiefly in the abdominal extremities; and uttered some plaintive cries.

At half after three he found great difficulty in moving himself; he dropped frequently on his hind feet, and his respiration was very much confined. He attempted several times to go through the wall, which shewed a sort of delirium. He had then a trembling in all his muscles.

At a quarter after four he lay down, and appeared in a profound sleep. His pulse was too quick to be counted.

At half after four he vomited up the paste he had taken; and some time after he rose, but walked with difficulty, falling sometimes on one side, sometimes on his hind legs.

He carried his head very low, his eyelids drooped, and he no longer distinguished objects; at least he struck himself against the walls and furniture of the laboratory in walking. His nostrils were scarcely sensible to the vapor of ammonia; and his ears heard nothing, for the most sudden noise did not make him stir.

He had not lost his memory however; for having put him, with a view to give him some vinegar and water, into the same posture as when he took the paste, he flew into a dreadful rage, as if all his strength had been at once renewed. From that time the symptoms he had experienced imperceptibly diminished, and about eight o'clock at night he had recovered all his outward senses, but was still greatly fatigued. The next day he ate as usual.

Such are the phenomena this animal exhibited: and every one must perceive in them the effects of narcotism and intoxication carried to their extreme; whence resulted a sort of delirium. It is probable, that, if he had not brought up the greater part of the matter, before it had time to produce its effect, it would have killed him.—*Ann. de Chim. vol.* lxxii. *p.* 53.

CRITICAL

